# Interdisciplinarity and Impact: Distinct Effects of Variety, Balance, and Disparity

**DOI:** 10.1371/journal.pone.0127298

**Published:** 2015-05-22

**Authors:** Jian Wang, Bart Thijs, Wolfgang Glänzel

**Affiliations:** Center for R&D Monitoring (ECOOM) and Department of Managerial Economics, Strategy and Innovation, University of Leuven, Leuven, Belgium; University of Illinois-Chicago, UNITED STATES

## Abstract

Interdisciplinary research is increasingly recognized as the solution to today’s challenging scientific and societal problems, but the relationship between interdisciplinary research and scientific impact is still unclear. This paper studies the association between the degree of interdisciplinarity and the number of citations at the paper level. Different from previous studies compositing various aspects of interdisciplinarity into a single indicator, we use factor analysis to uncover distinct dimensions of interdisciplinarity corresponding to variety, balance, and disparity. We estimate Poisson models with journal fixed effects and robust standard errors to analyze the divergent relationships between these three factors and citations. We find that long-term (13-year) citations (1) increase at an increasing rate with variety, (2) decrease with balance, and (3) increase at a decreasing rate with disparity. Furthermore, interdisciplinarity also affects the process of citation accumulation: (1) although variety and disparity have positive effects on long-term citations, they have negative effects on short-term (3-year) citations, and (2) although balance has a negative effect on long-term citations, its negative effect is insignificant in the short run. These findings have important implications for interdisciplinary research and science policy.

## Introduction

Interdisciplinary research has been increasingly viewed as the remedy for challenging contemporary scientific and societal problems. The National Academies [1] defined that “[i]nterdisciplinary research (IDR) is a mode of research by teams or individuals that integrates information, data, techniques, tools, perspectives, concepts, and/or theories from two or more disciplines or bodies of specialized knowledge to advance fundamental understanding or to solve problems whose solutions are beyond the scope of a single discipline or area of research practice.” As important ideas often transcend the scope of a single discipline, interdisciplinary research is the key to accelerating scientific discoveries and solving societal problems.

Given the normative interest in and the policy push for interdisciplinary research, it is important to investigate the drivers and consequences of interdisciplinary research. For example, Chavarro et al. [2] found that interdisciplinary papers have a greater emphasis on local issues. Rafols et al. [3] showed that multidisciplinary institutions are disadvantaged in discipline-based evaluation systems, but Millar [4] demonstrated a positive effect of interdisciplinary dissertation research on career placement and publication productivity of doctoral graduates. Bibliometric studies have also explored the relationship between interdisciplinary research and the citation impact, but findings are mixed. For example, Steele and Stier [5] found a positive effect of interdisciplinarity on citations for environmental sciences papers, where interdisciplinarity was measured as the disciplinary diversity of the cited references. Rinia et al. [6] studied physics programs in the Netherlands and operationalized interdisciplinarity as the ratio of non-physics publications. They found significantly negative correlations between interdisciplinarity and non-normalized citation-based metrics, but correlations became insignificant when field-normalization took place. Levitt and Thelwall [7] found that interdisciplinary papers received fewer citations in life and physical sciences but not in social sciences, where interdisciplinary papers were defined as papers published in journals assigned to multiple subject categories. Larivière and Gingras [8] analyzed all the Web of Science (WoS) articles published in 2000, measured interdisciplinarity as the percentage of its references to other disciplines, and found an inverted *U*-shaped relationship between interdisciplinarity and citations.

One possible explanation for these conflicting results is that they used different measures for interdisciplinarity. On the one hand, a number of interdisciplinarity indicators have been proposed, at different levels (e.g., paper, journal, institution, and fields) and using various bibliometric information (e.g., disciplinary memberships of authors, published journals, or cited references). On the other hand, the concept of interdisciplinarity remains abstract and complex; easy to intuit but difficult to define or measure [9]. One useful conceptualization is to view interdisciplinarity as the diversity of disciplines invoked in the research [10–12]. Furthermore, diversity has three distinct components [11]:

*Variety is the number of categories into which system elements are apportioned*. *It is the answer to the question*: *‘how many types of thing do we have*?*’*

*Balance is a function of the pattern of apportionment of elements across categories*. *It is the answer to the question*: *‘how much of each type of thing do we have*?*’*

*Disparity refers to the manner and degree in which the elements may be distinguished*. *It is the answer to the question*: *‘how different from each other are the types of thing that we have*?*’*

Many studies have devoted to compositing all aspects of interdisciplinarity into one single indicator. However, this paper adopts an opposite approach. We decompose different components of interdisciplinarity and explore their unique relationships with citations, at the individual paper level. Given that interdisciplinarity is an abstract and multidimensional concept, there might not be a straightforward answer to the question of whether interdisciplinary research draws higher impact. Instead, we should ask a different question: what kinds of interdisciplinarity have positive/negative associations with citations? In addition, nuanced understanding of the divergent dynamics underlying interdisciplinarity is important for informing interdisciplinary research and science policy.

In addition to the relationship between interdisciplinarity and long-term citations, we are also interested in the association between interdisciplinarity and the process of citation accumulation. Previous literature has long explored the process of citation ageing and reception, which is affected by a number of paper characteristics and social factors [13–16]. One intriguing phenomenon is delayed recognition or sleeping beauty, where a paper is uncited for a long time and then suddenly takes off and becomes highly cited [16–21]. In addition, scholars have also observed that citations to work in a different discipline have a longer delay than citations to work in the same discipline [22, 23], suggesting that interdisciplinary research is more likely to encounter citation delays. This hypothesis will be tested in this paper, in order to better understand the dynamic diffusion and recognition process of interdisciplinary work.

## Materials and Methods

We analyzed all the journal articles published in 2001 indexed in the Thomson Reuters Web of Science Core Collection (WoS). Only articles were analyzed, while all other document types such as reviews and letters were excluded. The year 2001 was chosen so that the studied papers could have a sufficiently long time window to accumulate their citations [14].

### Interdisciplinarity measures

Following previous literature, we constructed interdisciplinarity measures for each articles based on the disciplinary profile of its references, since referencing to prior literature in various disciplines indicates drawing and integrating knowledge pieces from these disciplines. Specifically, we constructed interdisciplinarity measures based on the WoS subject categories (SCs) referenced by each article. Interdisciplinarity measures constructed in this paper are listed in Table 1, which have been commonly used in the literature.

**Table 1:** Interdisciplinarity Measures.

Measure	Description
**Ratio of references to other subject categories**	
**Number of referenced subject categories**	*n*
**1—Gini**	$1-\frac{\sum \left(2i-n-1\right)x_{i}}{n\sum x_{i}}$, where *i* is the index, *x*_*i*_ is the number of references to the *i*-th subject category, and subject categories are sorted by *x*_*i*_ in non-decreasing order.
**Simpson index**	$1-\sum p_{i}^{2}$, where *p*_*i*_ = *x*_*i*_/*X*, and *X* = ∑*x*_*i*_
**Shannon entropy**	− ∑*p*_*i*_ log(*p*_*i*_)
**Average dissimilarity between referenced subject categories**	$\frac{1}{n\left(n-1\right)}\sum_{i\neq j}d_{ij}$, where *d*_*ij*_ is the dissimilarity between subject category *i* and *j*. Specifically, *d*_*ij*_ = 1 − *s*_*ij*_, where *s*_*ij*_ is the cosine similarity between subject category *i* and *j* based on their co-citation matrix.
**Rao-Stirling diversity**	$\sum_{i\neq j}p_{i}p_{j}d_{ij}$

The ratio of references which do not have any common subject categories with the focal paper (*ratio oth-disc refs*) has been used since a long time to capture the activity of borrowing knowledge from other disciplines [8, 24]. In addition, interdisciplinarity is often interpreted as disciplinary diversity, as an analogy to ecological diversity. The number of species has been used as a measure of species diversity [25], and membership to multiple fields has been used to indicate interdisciplinarity at the journal level [7, 26]. Similarly, at the paper level, the number of referenced subject categories can be used to indicate the richness and variety of disciplines invoked in the focal paper. The Gini index was originally proposed to measure income inequality, and has been used to capture the inequality, unevenness, and unbalance of the distribution of references across involved disciplines [27]. Note that a larger Gini indicates a lower level of diversity, so we used *1–Gini* in our analysis, which would have the same direction as diversity/interdisciplinarity. In addition, Shannon entropy [28] and Simpson index [29] are both popular diversity indices in the ecological literature and more recently the interdisciplinarity literature [9]. They capture both variety and balance of referenced disciplines. The original Simpson index is formulated as $\sum p_{i}^{2}$, which is also negatively associated with diversity. The formula adopted here, $1-\sum p_{i}^{2}$, is positively associated with diversity and is also referred to as Simpson index or Gini-Simpson index in the literature. Furthermore, while previous diversity literature focused on two dimensions of diversity (i.e., variety and balance), Rao [30] and Stirling [10] proposed a third dimension, that is, disparity. The average dissimilarity between referenced subject categories focuses on the disparity between invoked disciplines, and the Rao-Stirling diversity is a composite measure of all the three diversity components: variety, balance, and disparity [3, 11, 12].

Because the last two interdisciplinarity measures cannot be constructed when the focal article references fewer than two subject categories, we excluded these articles from the analysis. Nevertheless, regressions using the whole dataset for the other measures yielded consistent results. In total, our data have 646,669 papers. Descriptive statistics and Spearman correlations are reported in Table 2.

**Table 2:** Descriptive Statistics and Spearman Correlations (*N* = 646669).

	vars	mean	sd	min	max	1	2	3	4	5	6	7
**1**	Refs	15.03	12.59	1	615							
**2**	Referenced SCs	6.33	3.63	2	43	.69						
**3**	Ratio oth-disc refs	0.49	0.31	0	1	.09	.29					
**4**	1—Gini	0.67	0.16	0.15	1	-.81	-.57	.09				
**5**	Simpson	0.68	0.15	0.03	0.95	.36	.84	.42	-.09			
**6**	Shannon	1.43	0.51	0.07	3.22	.49	.94	.38	-.29	.97		
**7**	Avg dissimilarity	0.76	0.10	0.23	1	-.08	.16	.06	.01	.18	.19	
**8**	Rao-Stirling	0.25	0.07	0.01	0.44	.11	.64	.36	.05	.83	.79	.57

Data sourced from Thomson Reuters Web of Science Core Collection.

### Factor analysis

We used factor analysis to uncover components underlying these interdisciplinarity measures. The first step was to determine the number of factors to retain. A classic approach is Kaiser’s eigenvalue greater than one rule [31]. The idea is that the retained factor should explain more variance than the original standardized variables. Horn’s parallel analysis modifies Kaiser’s rule, where the criterion for each eigenvalue is different and also superior to one, and these criteria are obtained from a Monte-Carlo simulation [32]. Cattell’s scree test provides a graphical strategy, plotting the eigenvalues against the component numbers and searching for the elbow point [33]. However it does not yield a definitive number of factors to retain, which still relies on subjective judgments of the researcher. Recently, Raiche et al. [34] developed numerical solutions for Cattell’s scree test: (1) the optimal coordinate solution for the location of the scree and (2) the acceleration factor solution for the location of the elbow. We implemented all these methods.

After determining the number of factors to retain, we extracted these factors using the varimax rotated principal components method. Specifically, we used the *principal* function in the *R* package *psych*. In addition, the number of referenced subject categories is highly skewed, so its nature logarithm was used in the factor analysis.

### Regression analysis

To study the relationship between interdisciplinarity and long-term citations at the article level, we ran regressions, using the number of long-term citations (in a 13-year time window from 2001 to the end of 2013) as the dependent variable and interdisciplinarity measures and extracted factors as explanatory variables. To explore the association between interdisciplinarity and citation delay, we further estimated the effects of interdisciplinarity on short-term citations (in a 3-year time window from 2001 to 2003) and compared them with the effects on long-term citations. In addition, we also adopted another dependent variable, *citation delay*, to describe how slow a paper accumulates its citations. Wang [14] developed a *citation speed* measure:

$$Citation\;Speed=\frac{\sum_{1}^{n-1}C_{i}/C_{n}}{n-1}$$

where *n* is the total number of years (i.e., 13), and *C*_*i*_ the cumulative number of citations by the *i*-th year. Since the cumulative citation ratio is monotonically non-decreasing, a paper accumulates its citations fast would rise early and then stay at the high level, so it would have a high value of *citation speed*. This measure takes value between 0 and 1. Since our theoretical discussion is about citation delay, we simply define:

$$Citation\;Delay=1-Citation\;Speed=1-\frac{\sum_{1}^{n-1}C_{i}/C_{n}}{n-1}$$

Because this ratio-based measure might not be very reliable when the denominator is too small, so when running regressions using this measure as the dependent variable, we excluded papers with fewer than 12 citations (which is the median value in our data). Nevertheless, results are robust if we relax this restriction.

For all our regressions, we incorporated journal fixed effects to control for (1) unobserved topic or subfield heterogeneities at the fine-grained journal level and (2) journal reputation effects [35]. Therefore, we estimated the within-journal effects, in other words, we were evaluating the association between interdisciplinarity and citations among papers published in the same journal. In addition, the following variables were incorporated as controls: the number of authors, the number of countries, the number of pages, and the number of references. These variables have been observed to be related to citations [36–38]. The numbers of authors, pages, and references are skewed so that their natural logarithms were used in regression analyses. The number of countries is still highly skewed after logarithm transformation, so we created a dummy variable, *international*: 1 if the paper has authors from more than one country, and 0 otherwise. In our sample, about 19% of the papers are internationally coauthored.

We estimated fixed effects least squares models for *citation delay*, which is roughly normally distributed. Specifically, we implemented the *xtreg* function in *STATA*, which is equivalent to a standard OLS regression with a complete set of journal dummies. In addition, robust standard errors were clusters at journals. When analyzing the number of long- and short-term citations, the fixed-effects Poisson models with robust standard errors were estimated. Because citation counts are over-dispersed count variables, we used Poisson regression with robust standard errors, following previous literature [39–42]. An alternative is the negative binomial model. However, because the Poisson model is in the linear exponential class, Gourieroux et al. [43] have shown that the Poisson estimator and the robust standard errors are consistent so long as the mean is correctly specified even under misspecification of the distribution, but the negative binomial estimator is inconsistent if the true underlying distribution is not negative binomial. Therefore, we adopted the Poisson model with robust standard errors for our empirical analysis. Furthermore, we incorporated journal fixed effects. Such fixed effects Poisson models can be fitted by conditioning out the individual fixed effects [44]. Specifically, we used the *xtpoisson* command in *STATA*, which implements the formula presented in Wooldridge [45].

## Results

### Decomposing interdisciplinarity

**Fig 1:**
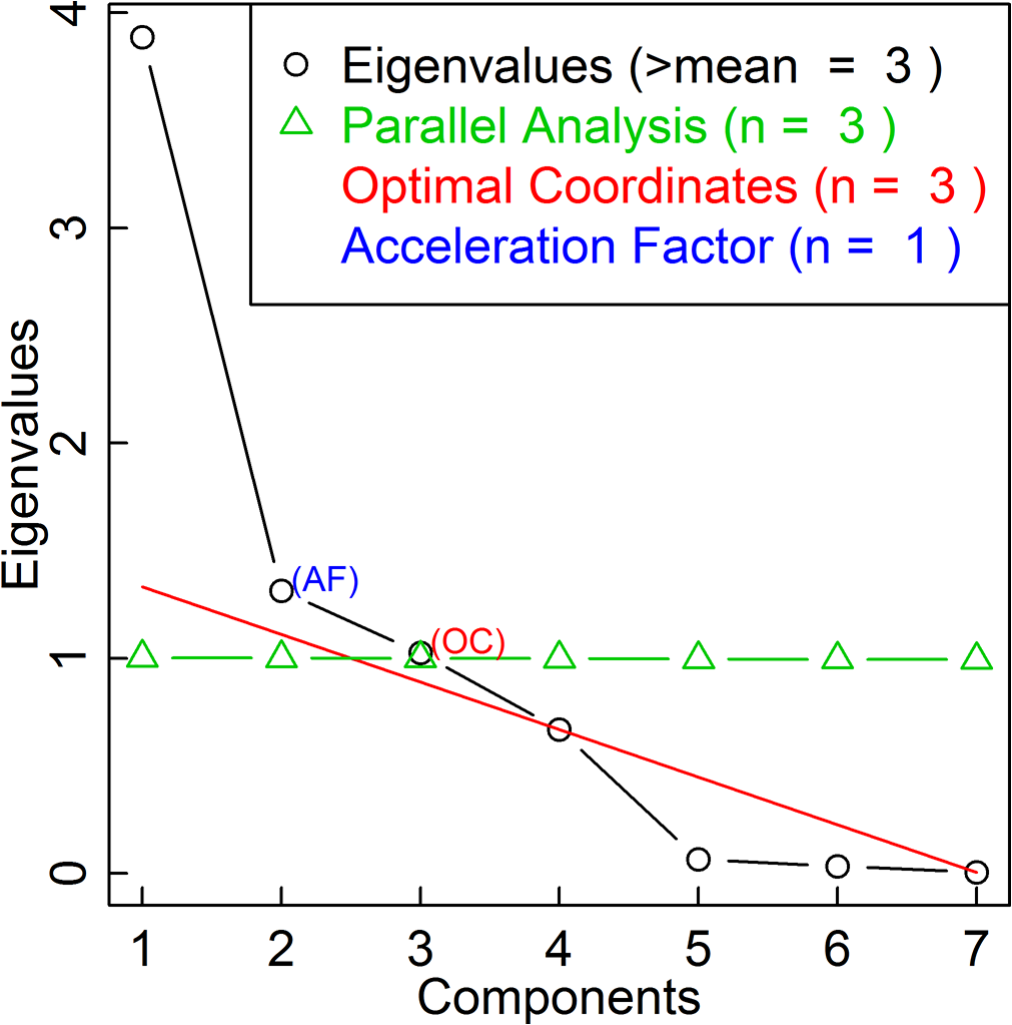
Determining the Number of Factors. Data sourced from Thomson Reuters Web of Science Core Collection.

We used the following variables in the factor analysis: log number of referenced subject categories, ratio of references to other subject categories, *1–Gini*, Simpson index, Shannon entropy, average dissimilarity between referenced subject categories, and Rao-Stirling diversity. As plotted in Fig 1, the first three eigenvalues are greater than one, so three factors should be retained according to Kaiser’s rule. Different criteria for different eigenvalues based on Horn’s parallel analysis are also plotted (triangles), which form a downward slopped curve. The conclusion is also three factors. Raiche’s nongraphic solutions for Cattell’s scree test lead to conflicting conclusions: the optimal coordinate approach suggests three factors, while the acceleration factor approach suggests one factor to retain. Considering (1) the consensus between the classic Kaiser’s rule and Horn’s parallel analysis, (2) the divergence in this recent nongraphic solution for Cattell’s scree test, and (3) that the optimal coordinate solution actually agrees with the more conventional approaches. We decided to retain three factors.

Subsequently, we extracted three factors using the varimax rotated principal components method, and the cumulative proportion variance explained is 0.89. Factor loadings are reported in Table 3. Simpson index and Shannon entropy have the highest loading on the first factor, which reflects the variety aspect of disciplinary diversity. *1–Gini* has the highest loading on the second factor, which reflects balance, and the average dissimilarity between referenced subject categories has the highest loading on the third factor, which reflects disparity. The results are also in line with Harrison and Klein [46], which suggested Simpson index and Shannon entropy as measures of variety, and Gini as a measure of unbalance. Note that in Harrison and Klein referred Simpson index as Blau index, Shannon entropy as Teachman entropy, and unbalance as disparity.

**Table 3:** Factor Loading.

	Factor 1	Factor 2	Factor 3
**ln(referenced SCs)**	0.78	-0.59	0.15
**Ratio oth-disc refs**	0.67	0.35	-0.17
**1–Gini**	-0.07	0.94	0.05
**Simpson**	0.93	-0.11	0.18
**Shannon**	0.91	-0.32	0.18
**Avg dissimilarity**	0.09	0.00	0.95
**Rao-Stirling**	0.77	0.04	0.59

Cumulative proportion variance explained: 0.89.

Data sourced from Thomson Reuters Web of Science Core Collection.

### Interdisciplinarity and long-term impact

**Fig 2:**
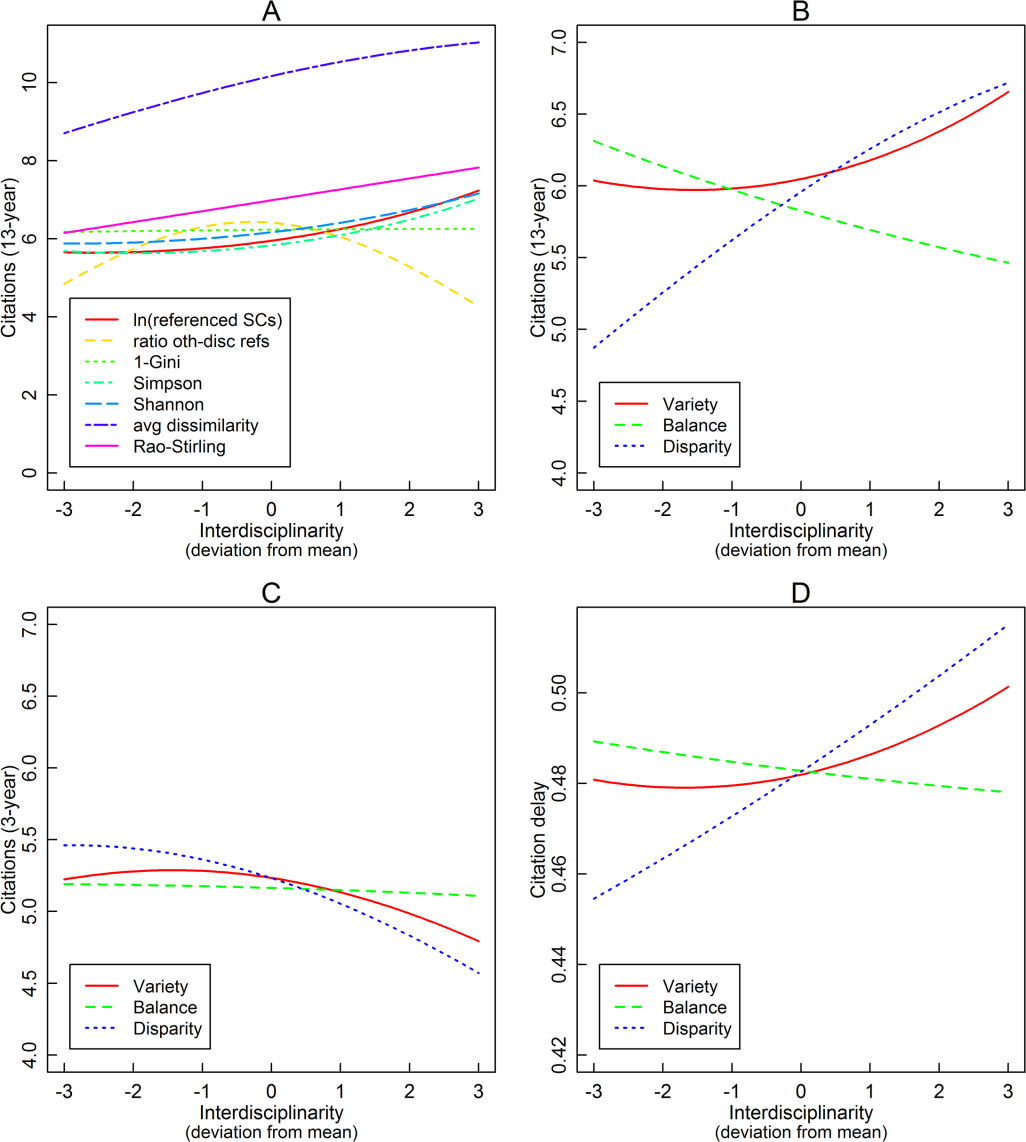
Interdisciplinarity and Citations. Data sourced from Thomson Reuters Web of Science Core Collection.

We first estimated fixed effects Poisson models using the long-term citation counts as the dependent variable and original interdisciplinarity measures as independent variables (Table 4). For each interdisciplinarity measure, we first fitted a model with the linear term and subsequently added the squared term to test its potential non-linear relationship with long-term citations. Taking Rao-Stirling diversity as an example (column 13), holding that the papers are published in the same journal, with the same number of authors, pages, and references, and have the same status in terms of whether being internationally coauthored, the number of expected long-term citations increases by 58% as Rao-Stirling diversity increases by 1, (the theoretical minimum and maximum values are 0 and 1 respectively, and the observed minimum and maximum values are 0.01 and 0.44 respectively). Furthermore, as shown in column 14, the quadratic term is insignificant, while the linear term is still significantly positive. The estimated citations by each original interdisciplinarity measure are also plotted in Fig 2A for a better visual inspection. These plots are based on models with both linear and quadratic terms (i.e., column 2, 4, 6, 8, 10, 12, and 14), holding the log number of authors, pages, and references at their means, *international* at 0, and journal fixed effect being 0. Four types of relationship with citations are observed: (1) The log number of referenced subject categories, Simpson index, and Shannon entropy have a positive relationship with the number of citations, in line with Steele and Stier [5]. Furthermore, long-term citations increase with these variables at an increasing rate. (2) Average dissimilarity and Rao-Stirling diversity also have a positive relationship with long-term citations, but citations increases with them at a decreasing rate (although insignificant). (3) The ratio of references to other subject categories has an inverted *U*-shaped relationship with citations, in line with Larivière and Gingras [8]. (4) *1–Gini* has an insignificant relationship with long-term citations. However, this is because of its high correlation with the number of references. If we drop the number of references from the regression, then we would observe a strong negative effect of *1–Gini* on citations. The divergent results suggest that the low consensus in previous literature regarding the relationship between interdisciplinarity and citations may be partially explained by their different choices of the interdisciplinarity measure.

**Table 4:** Fixed Effects Poisson Models with Original Interdisciplinarity Measures (*N* = 646223).

	Long-term citations (13-year)													
	(1)	(2)	(3)	(4)	(5)	(6)	(7)	(8)	(9)	(10)	(11)	(12)	(13)	(14)
ln(authors)	0.16***	0.16***	0.16***	0.16***	0.16***	0.16***	0.16***	0.16***	0.16***	0.16***	0.16***	0.16***	0.16***	0.16***
	(0.01)	(0.01)	(0.01)	(0.01)	(0.01)	(0.01)	(0.01)	(0.01)	(0.01)	(0.01)	(0.01)	(0.01)	(0.01)	(0.01)
International	0.00	0.00	0.00	0.00	0.00	0.00	0.00	0.00	0.00	0.00	0.00	0.00	0.00	0.00
	(0.01)	(0.01)	(0.01)	(0.01)	(0.01)	(0.01)	(0.01)	(0.01)	(0.01)	(0.01)	(0.01)	(0.01)	(0.01)	(0.01)
ln(pages)	0.40***	0.40***	0.40***	0.40***	0.40***	0.40***	0.41***	0.41***	0.41***	0.41***	0.40***	0.40***	0.40***	0.40***
	(0.03)	(0.03)	(0.03)	(0.03)	(0.03)	(0.03)	(0.03)	(0.03)	(0.03)	(0.03)	(0.03)	(0.03)	(0.03)	(0.03)
ln(refs)	0.27***	0.27***	0.31***	0.30***	0.31***	0.31***	0.30***	0.30***	0.29***	0.29***	0.30***	0.30***	0.30***	0.30***
	(0.01)	(0.01)	(0.01)	(0.01)	(0.01)	(0.01)	(0.01)	(0.01)	(0.01)	(0.01)	(0.01)	(0.01)	(0.01)	(0.01)
ln(referenced SCs)	0.08***	-0.01												
	(0.01)	(0.04)												
(ln(referenced SCs))^2^		0.02*												
		(0.01)												
Ratio oth-disc refs			-0.08***	0.32***										
			(0.02)	(0.05)										
(Ratio oth-disc refs)^2^				-0.40***										
				(0.04)										
1-Gini					0.01	0.03								
					(0.03)	(0.17)								
(1-Gini)^2^						-0.01								
						(0.12)								
Simpson							0.18***	-0.30*						
							(0.04)	(0.12)						
Simpson^2^								0.40***						
								(0.11)						
Shannon									0.07***	0.00				
									(0.01)	(0.03)				
Shannon^2^										0.02^+^				
										(0.01)				
Avg dissimilarity											0.43***	1.02**		
											(0.07)	(0.35)		
(Avg dissimilarity)^2^												-0.41		
												(0.25)		
Rao-Stirling													0.58***	0.65*
													(0.08)	(0.29)
(Rao-Stirling)^2^														-0.16
														(0.57)
Journal fixed effects	YES	YES	YES	YES	YES	YES	YES	YES	YES	YES	YES	YES	YES	YES
Log pseudolikelihood	-8637695	-8637136	-8641587	-8635054	-8644074	-8644073	-8640234	-8639142	-8638382	-8638061	-8635233	-8635032	-8635015	-8635008
*χ* ^2^	3098***	3228***	3001***	3698***	2941***	3174***	3005***	3092***	3050***	3053***	4083***	4066***	3903***	4175***

Cluster-robust standard errors in parentheses.

*** p<.001

** p<.01

* p<.05

^+^ p<.10.

Data sourced from Thomson Reuters Web of Science Core Collection.

Table 5 reports fixed effects Poisson models using the extracted interdisciplinarity factors as independent variables. Variety, balance, and disparity are the three extracted factors, and they follow the standard normal distribution with mean equals to 0 and standard deviation equals to 1. Holding that the papers are published in the same journal, with the same number of authors, pages and references, and have the same status in terms of whether being internationally coauthored, the expected number of long-term citations increases by 1.48% as variety increases by 1 standard deviation (column 1), decreases by 2.45% as balance increases by 1 standard deviation (column 3), and increases by 5.77% as disparity increases by 1 standard deviation. Squared terms are subsequently added to test the non-linearity in these relationships. On the one hand, the square terms of variety and disparity are significant, suggesting nonlinear relationships. On the other hand, the squared term of balance is insignificant, suggesting a simply linear relationship. Fig 2B plots the estimated long-term citations with variety, balance, and disparity, based on column 2, 4, and 6 of Table 5. Again, for these estimations, we fix journal fixed effect at 0, *international* at 0, and all other variables at their means.

**Table 5:** Fixed Effects Poisson Models: Interdisciplinarity and Long-Term Impact (*N* = 646223).

	Long-term citations (13-year)							
	(1)	(2)	(3)	(4)	(5)	(6)	(7)	(8)
ln(authors)	0.1588***	0.1586***	0.1600***	0.1600***	0.1590***	0.1586***	0.1578***	0.1575***
	(0.0105)	(0.0105)	(0.0106)	(0.0106)	(0.0110)	(0.0110)	(0.0107)	(0.0107)
International	-0.0009	-0.0008	-0.0013	-0.0013	-0.0025	-0.0025	-0.0023	-0.0022
	(0.0130)	(0.0130)	(0.0130)	(0.0130)	(0.0135)	(0.0135)	(0.0133)	(0.0133)
ln(pages)	0.4054***	0.4055***	0.4022***	0.4019***	0.3958***	0.3963***	0.3965***	0.3965***
	(0.0295)	(0.0295)	(0.0295)	(0.0294)	(0.0301)	(0.0302)	(0.0300)	(0.0300)
ln(refs)	0.3021***	0.3013***	0.2868***	0.2871***	0.3056***	0.3045***	0.2855***	0.2836***
	(0.0078)	(0.0077)	(0.0105)	(0.0105)	(0.0082)	(0.0083)	(0.0118)	(0.0119)
Variety	0.0148*	0.0162*					0.0137^+^	0.0154^+^
	(0.0061)	(0.0064)					(0.0078)	(0.0083)
Variety^2^		0.0052*						0.0044^+^
		(0.0026)						(0.0026)
Balance			-0.0245**	-0.0241**			-0.0194^+^	-0.0194^+^
			(0.0074)	(0.0073)			(0.0106)	(0.0108)
Balance^2^				0.0009				0.0021
				(0.0033)				(0.0030)
Disparity					0.0577***	0.0535***	0.0528***	0.0488***
					(0.0075)	(0.0074)	(0.0088)	(0.0087)
Disparity^2^						-0.0045^+^		-0.0036
						(0.0025)		(0.0025)
Journal fixed effects	YES	YES	YES	YES	YES	YES	YES	YES
Log pseudolikelihood	-8642990	-8642683	-8642595	-8642588	-8629711	-8629503	-8628738	-8628365
*χ* ^2^	2946***	2957***	2967***	2961***	4450***	4438***	4552***	4807***

Cluster-robust standard errors in parentheses.

*** p<.001

** p<.01

* p<.05

^+^ p<.10.

Data sourced from Thomson Reuters Web of Science Core Collection.

We observe that long-term citations increase at an increasing rate with variety, which is in line with the information processing perspective, which suggests that cognitive variety is very important for creative and innovative work [47–49]. For interdisciplinary research, integrating knowledge from more disciplines contributes to potentially more broadly useful outcomes.

We also observe a negative relationship between balance and long-term citations, which concur with Uzzi et al. [50], which has shown that that a paper with both higher levels of novelty and conventionality is more likely to be a top cited paper. In other words, a paper is more likely to be highly cited if it is embedded in the core of a discipline (drawing most of its prior knowledge and references from one discipline) and at the same time borrows knowledge from some remote disciplines. Therefore, balanced interdisciplinary paper without a disciplinary core is relatively less cited. However, the mechanism underlying this negative association between long-term citations and balance is still unclear. On the one hand, it could be that interdisciplinary research driven evenly by different disciplinary logics is more likely to fail, in the process of integrating these logics into something useful. Therefore, having one disciplinary core and simultaneously borrowing knowledge from other disciplines is a more effective research strategy, compared with drawing knowledge evenly from multiple disciplines. On the other hand, it could be that the current science system is biased against balanced interdisciplinary research. There are anecdotes that balanced interdisciplinary research truly transcend disciplinary boundaries, but is difficult to evaluate and more likely to be unnoticed, simply because most scientists are trained within a discipline and unable to recognize its value, although such balanced interdisciplinary research is very novel and potentially broadly useful.

In addition, we observe that long-term citations increase with disparity but at a decreasing rate. This is in line with the combinatorial novelty literature that combining more distant disciplines is more novel than combining neighboring disciplines [49, 50]. Furthermore, there is a complex connection between novelty and impact. On the one hand, novelty is important for generating impact. On the other hand, a highly novel paper might not be useful for other scientists’ work and therefore fail to generate high impact [51–53]. We do observe that that the marginal return from disparity is decreasing. It’s possible that the effect of disparity on long-term citations may turn into a negative one after certain point, but this threshold is about six standard deviations above the mean, which is beyond the maximum disparity value in our data.

### Interdisciplinarity and citation delay

The preceding section demonstrates that variety, balance, and disparity have distinct relationships with long-term citations, and this section investigates how interdisciplinarity affects the process of citation accumulation. First, we estimated fixed effects Poisson models using the short-term citations as the dependent variable (Table 6). Variety and disparity have significantly negative effects on short-term citations, while balance has no significant effects. Therefore, although variety and disparity contribute to a higher impact in the long run, their positive effects take time to show. On the contrary, they lead to lower citation impact in the short run. In addition, although balance lead to lower impact in the long run, its disadvantage also takes time to show and is unobservable in the short run.

**Table 6:** Fixed Effects Poisson Models: Interdisciplinarity and Short-Term Impact (*N* = 644956).

	Short-term citations (3-year)							
	(1)	(2)	(3)	(4)	(5)	(6)	(7)	(8)
ln(authors)	0.2013***	0.2015***	0.2002***	0.2001***	0.2011***	0.2007***	0.2018***	0.2016***
	(0.0142)	(0.0142)	(0.0142)	(0.0142)	(0.0142)	(0.0142)	(0.0140)	(0.0140)
International	0.0095	0.0094	0.0097	0.0097	0.0102	0.0102	0.0100	0.0099
	(0.0142)	(0.0142)	(0.0141)	(0.0141)	(0.0138)	(0.0139)	(0.0138)	(0.0138)
ln(pages)	0.2501***	0.2500***	0.2512***	0.2512***	0.2548***	0.2554***	0.2536***	0.2545***
	(0.0284)	(0.0284)	(0.0284)	(0.0283)	(0.0288)	(0.0288)	(0.0286)	(0.0286)
ln(refs)	0.3795***	0.3802***	0.3736***	0.3735***	0.3760***	0.3747***	0.3757***	0.3768***
	(0.0079)	(0.0079)	(0.0093)	(0.0091)	(0.0082)	(0.0083)	(0.0104)	(0.0106)
Variety	-0.0130*	-0.0143*					-0.0088	-0.0116
	(0.0054)	(0.0057)					(0.0075)	(0.0080)
Variety^2^		-0.0050*						-0.0043^+^
		(0.0020)						(0.0023)
Balance			-0.0024	-0.0026			-0.0037	-0.0022
			(0.0072)	(0.0075)			(0.0115)	(0.0121)
Balance^2^				-0.0003				-0.0016
				(0.0034)				(0.0030)
Disparity					-0.0237*	-0.0297**	-0.0229*	-0.0283*
					(0.0094)	(0.0095)	(0.0116)	(0.0113)
Disparity^2^						-0.0051*		-0.0056*
						(0.0021)		(0.0023)
Journal fixed effects	YES	YES	YES	YES	YES	YES	YES	YES
Log pseudolikelihood	-1992283	-1992235	-1992423	-1992423	-1991992	-1991944	-1991906	-1991818
*χ* ^2^	3981***	3996***	3933***	4149***	5223***	5198***	5414***	5519***

Cluster-robust standard errors in parentheses.

*** p<.001

** p<.01

* p<.05

^+^ p<.10.

Data sourced from Thomson Reuters Web of Science Core Collection.

Table 7 reports fixed effects least squares models with *citation delay* as the dependent variable. Both variety and disparity have a significantly positive relationship with citation delay, indicating that interdisciplinary papers with a higher level of variety or disparity are more likely to encounter citation delay, being relatively less cited in the short run but highly cited in the long run. In addition, balance has a negative relationship with citation delay, meaning that interdisciplinary papers with a higher level of balance is less likely to encounter citation delay. This is because these papers have an early rise and early decline in their process of citation accumulation, that is, they receive their limited number of citation in the short run and then quickly cease to be cited.

**Table 7:** Fixed Effects Least Squares Models: Interdisciplinarity and Citation Delay (*N* = 332649).

	Citation delay							
	(1)	(2)	(3)	(4)	(5)	(6)	(7)	(8)
ln(authors)	-0.0054***	-0.0055***	-0.0052***	-0.0052***	-0.0053***	-0.0053***	-0.0055***	-0.0055***
	(0.0005)	(0.0005)	(0.0005)	(0.0005)	(0.0005)	(0.0005)	(0.0005)	(0.0005)
International	-0.0011*	-0.0011*	-0.0012**	-0.0012**	-0.0013**	-0.0013**	-0.0013**	-0.0013**
	(0.0004)	(0.0004)	(0.0004)	(0.0004)	(0.0004)	(0.0004)	(0.0004)	(0.0004)
ln(pages)	0.0158***	0.0158***	0.0154***	0.0153***	0.0140***	0.0140***	0.0143***	0.0141***
	(0.0009)	(0.0009)	(0.0009)	(0.0009)	(0.0009)	(0.0009)	(0.0009)	(0.0009)
ln(refs)	-0.0188***	-0.0190***	-0.0194***	-0.0194***	-0.0183***	-0.0182***	-0.0199***	-0.0202***
	(0.0004)	(0.0004)	(0.0005)	(0.0005)	(0.0003)	(0.0004)	(0.0006)	(0.0006)
Variety	0.0030***	0.0034***					0.0021***	0.0027***
	(0.0003)	(0.0004)					(0.0004)	(0.0004)
Variety^2^		0.0010***						0.0009***
		(0.0002)						(0.0002)
Balance			-0.0019***	-0.0019***			-0.0011**	-0.0014**
			(0.0004)	(0.0004)			(0.0004)	(0.0004)
Balance^2^				0.0001				0.0004*
				(0.0002)				(0.0002)
Disparity					0.0099***	0.0101***	0.0094***	0.0095***
					(0.0004)	(0.0004)	(0.0004)	(0.0004)
Disparity^2^						0.0002		0.0004^+^
						(0.0002)		(0.0002)
Journal fixed effects	YES	YES	YES	YES	YES	YES	YES	YES
R^2^ within	0.0163	0.0166	0.0157	0.0157	0.0227	0.0227	0.0230	0.0232
F	563***	472***	549***	461***	696***	584***	501***	355***

Cluster-robust standard errors in parentheses.

*** p<.001

** p<.01

* p<.05

^+^ p<.10.

Data sourced from Thomson Reuters Web of Science Core Collection.

Results for the control variables might also be worth noting. Comparing papers published in the same journal with the same number of authors, pages, and references, internationally coauthored papers do not have significantly more citations than single country papers, in both the short run and the long run. However, they do have a lower citation delay score, indicating that being internationally coauthored does contribute to faster knowledge diffusion. In addition, the number of authors and references has significantly positive effects on both short- and long-term citations, and they also have negative effects on citation delay. It suggests that more authors or more references contribute to not only more but also faster citations. In other words, the benefit of more authors or more references is stronger in the short run and slowly declines over time, but still not completely fade away by the 13^th^ year after the paper is published. Furthermore, the number of pages has a slightly different influence on citations. The number of pages has positive effects on both short-term and long-term citations, but also a positive effect on citation delay. Therefore, the number of pages does not help to attract citations faster but has a more enduring effect. Its positive effect on citations strengthens over time.

### Robustness tests

We have done a number of analyses to test the robustness of our findings. Regressions of citation counts reported in this paper are based on the conditional fixed effects Poisson models with robust standard errors. We incorporated journal fixed effects to estimate within-journal effects, and such models can be estimated by a conditional maximum likelihood method which conditions out journal fixed effects [44, 45, 54]. Specifically, we implemented the *xtpoisson* function in *STATA* [55]. Given that the negative binomial models are also commonly used in the literature, we also tried the negative binomial models. Hausman et al. [44] also developed a conditional maximum likelihood strategy for negative binomial models, which is implemented in the *xtnbreg* function in *STATA* [56]. However, this method allows for individual-specific variation in the dispersion parameter rather than in the conditional mean, and therefore does not qualify as a true fixed effects method [57–59]. We fitted the *xtnbreg* models for a robustness check, note that some between-journal differences may remain in the estimates. We got consistent results, except that the effect of balance on short-term citations became significantly negative (which is insignificantly negative in Table 6). However, this inconsistency does not challenge our conclusions.

In addition, we used alterative measures to capture the speed of citation accumulation or citation delay: (1) the ratio between accumulative citation counts by year 3 and by year 13, and (2) the year when the paper gets 50% of its total citations [60]. Results are robust. Furthermore, we excluded papers with fewer than 12 (which is the median) citations from the regressions. We also ran regressions without such constraints and got consistent results.

Furthermore, journals are sometimes assigned to multiple subject categories in WoS. It is possible that a paper with only one reference would have two or more referenced subject categories, because this one reference is from a journal with multiple subject categories. This may cause problems for our interdisciplinarity measures. Therefore, we used the more aggregated ECOOM discipline (68 disciplines) classification scheme [61] instead of the WoS subject categories, since using more aggregated field classifications would reduce the instances of journals having multiple field assignments and therefore would mitigate the potential measurement issues. Results remained consistent. Another related issue pertains to multidisciplinary journals, since the disciplinary memberships of papers published in these journals are not clear. We excluded references in the *multidisciplinary sciences* subject category in our interdisciplinarity measure, and got consistent results.

In addition, because the average dissimilarity between referenced subject categories and Rao-Stirling diversity require at least two referenced subject categories, we excluded papers with fewer referenced subject categories from the analyses. Using the whole sample and running regressions for the rest interdisciplinarity measures, we also got consistent results.

However, as a bibliometric study, this paper cannot avoid some fundamental limitations in the bibliometric data, such as potential errors in the use of citations as a measure of scientific impact [62–64]. Please refer to [62] for a comprehensive discussion on the limitations and justifications. In addition, our interdisciplinarity measures are based on references in the scientific outputs and therefore cannot capture the knowledge integration in the interdisciplinary research process [9].

## Discussion

This paper studies three different aspects of interdisciplinarity and investigates their distinct relationships with citation impact and citation delay. The factor analysis extracts three components underlying various interdisciplinarity measures, and these three components correspond to variety, balance, and disparity. Regression analysis further uncovers their different relationships with long-term citations, specifically, long-term citations (1) increase at an increasing rate with variety, (2) decrease with balance, and (3) increase at a decreasing rate with disparity. Furthermore, although variety and disparity have positive effects on long-term citations, they have negative effects on short-term citations. In addition, although balance has a negative effect on long-term citations, such negative effect is insignificant in the short run.

This paper contributes to the interdisciplinarity literature and science policy. First, we advocate the idea of using different interdisciplinarity measures according to the specific context. This paper demonstrates that various interdisciplinarity measures bear non-identical relationships with scientific impact. Interdisciplinarity is an abstract and multidimensional concept, and different aspects of interdisciplinarity may (1) respond to certain individual, team, or institutional factors in distinct ways, and (2) have unique consequences in terms of usefulness or impact. Furthermore, various theories which may shed light on interdisciplinarity research have their own emphases. For example, the information processing perspective focuses on cognitive variety, while the combinatorial novelty literature emphasizes disparity. Therefore, it’s important to choose a suitable interdisciplinarity measure consistent with the invoked theory and focal research question.

Second, this paper suggests a more refined policy agenda for encouraging interdisciplinary research. This paper pushes forward the research on the relationship between interdisciplinarity and scientific impact: from a dichotomous question of whether interdisciplinary research draws higher impact towards a more complicated question about differentiated dynamics underlying interdisciplinarity. Answers to this more complicated question are important for more effective science policies. As science increasingly deals with boundary-spanning problems, various policy and funding initiatives have been developed to encourage interdisciplinary research, such as the US National Science Foundation (NSF) solicited interdisciplinary programs, the US National Institutes of Health (NIH) common fund’s interdisciplinary research program, European Research Council (ERC) synergy grants, and UK Research Councils’ cross-council funding agreement. However, interdisciplinarity is an abstract and multidimensional concept, and nuanced understanding of these different dimensions and their consequences are important for effective policies. Specifically, the positive relationship between variety and impact demonstrates the benefits of cognitive variety for creative work. Therefore, policy and funding initiatives can encourage research across more disciplinary boundaries and integrating knowledge from more disciplines. Furthermore, the positive relationship between disparity and impact also suggests potential improvements from encouraging interdisciplinary research across more remotely connected disciplines. However, since the positive marginal effect is decreasing, the policy might not want to push too far. It’s possible that the effect of disparity on citations turns into a negative one when disparity is too high, that is, integrating disciplines too far apart may fail to find a common ground for producing something useful. In addition, the negative relationship between balance and impact may suggest that the most effective interdisciplinary research strategy, in terms of generating impact, is to have one disciplinary core and simultaneously borrow knowledge from some other disciplines, rather than drawing knowledge evenly from multiple disciplines. It is possible that research driven evenly by different disciplinary logics fails to integrate these logics into something useful. On the other hand, this negative association might suggest that balanced interdisciplinary research is biased against in the current discipline-based science system, in which scientists are mostly trained within a single discipline and fail to realize the value of balanced interdisciplinary work which truly transcends disciplinary boundaries. However, further research is required in order to better understand this problem. Specifically, to claim the bias against balanced interdisciplinary research, we need to estimate the unbiased “should-be” scientific impact first and then compare it with the observed citations. To recommend policies encouraging unbalanced instead of balanced interdisciplinary research, we need to test the usefulness and value of the papers directly, instead of only examining citation counts.

Third, this paper suggests a longer citation time window for evaluating interdisciplinary research. Although variety and disparity have significantly positive effects on long-term citations, they have negative effects on short-term citations. Therefore, if we adopt a very short citation time window, we would systematically underestimate the impact of interdisciplinary papers with a higher level of variety or disparity. In addition, this paper also demonstrates that the dynamic process of citation accumulation is an important aspect to be investigated in interdisciplinarity research and other science studies, in addition to long-term citations.

## Supporting Information

S1 Data Data underlying the findings. (ZIP)
